# Physical Fitness and Frailty in Males after Allogeneic Hematopoietic Stem Cell Transplantation in Childhood: A Long-Term Follow-Up Study

**DOI:** 10.3390/cancers14143310

**Published:** 2022-07-07

**Authors:** Anu Suominen, Anu Haavisto, Sidsel Mathiesen, Malene Mejdahl Nielsen, Päivi M. Lähteenmäki, Kaspar Sørensen, Marianne Ifversen, Christian Mølgaard, Anders Juul, Klaus Müller, Kirsi Jahnukainen

**Affiliations:** 1 New Children’s Hospital, Pediatric Research Center, University of Helsinki and Helsinki University Hospital, 00014 Helsinki, Finland; kirsi.jahnukainen@ki.se; 2 Department of Psychology and Logopedics, University of Helsinki, 00014 Helsinki, Finland; anu.haavisto@helsinki.fi; 3 Department of Women’s and Children’s Health, Karolinska Institutet, 171 77 Stockholm, Sweden; paivi.maria.lahteenmaki@tyks.fi; 4 Department of Pediatrics and Adolescent Medicine, Copenhagen University Hospital—Rigshospitalet, 2100 Copenhagen, Denmark; sidsel.mathiesen@regionh.dk (S.M.); malene.mejdahl.nielsen@regionh.dk (M.M.N.); kaspar.soerensen.01@regionh.dk (K.S.); marianne.ifversen@regionh.dk (M.I.); 5 Department of Pediatrics and Adolescent Medicine, Turku University Hospital, 20521 Turku, Finland; 6 Department of Nutrition, Exercise and Sports, Copenhagen University Hospital—Rigshospitalet, 2100 Copenhagen, Denmark; christian.moelgaard@regionh.dk; 7 Pediatric Nutrition Unit, Copenhagen University Hospital—Rigshospitalet, 2100 Copenhagen, Denmark; 8 Department of Growth and Reproduction, Copenhagen University Hospital—Rigshospitalet, 2100 Copenhagen, Denmark; anders.juul@regionh.dk; 9 Institute for Inflammation Research, Copenhagen University Hospital—Rigshospitalet, 2100 Copenhagen, Denmark; klaus.mueller@regionh.dk; 10 Department of Clinical Medicine, Copenhagen University Hospital—Rigshospitalet, 2100 Copenhagen, Denmark; 11 NORDFERTIL Research Lab Stockholm, Department of Women’s and Children’s Health, Karolinska Institutet and University Hospital, 171 77 Stockholm, Sweden

**Keywords:** physical fitness, GvHD, frailty, aging, pediatric hematopoietic stem cell transplantation, late effects

## Abstract

**Simple Summary:**

The prevalence of frailty is increased among young adult childhood cancer survivors and is associated with early morbidity and mortality. The aim of our study was to analyze physical fitness, physical activity and the prevalence of frailty in male long-term survivors of pediatric allogeneic hematopoietic stem cell transplantation. We observed significantly lower muscle strength and muscle endurance in the hand-grip and sit-to-stand tests compared to the age and sex matched normative reference values of the tests. Furthermore, 30% of the survivors were considered pre-frail or frail. Chronic graft-versus-host disease, shorter stature, higher body fat mass and hazardous drinking predicted prefrail/frail status. Common cardiovascular risk factors were associated with poor physical fitness and low physical activity level. These results indicate a need for cardiometabolic follow up as well as health education in the decades following HSCT.

**Abstract:**

Purpose and methods: To analyze physical fitness, physical activity and the prevalence of frailty in male long-term survivors of pediatric allogeneic hematopoietic stem cell transplantation (HSCT). We performed a Nordic two-center study of 98 male survivors (mean age 28.7 years, range 18.5–47.0) treated with pediatric allogeneic hematopoietic stem cell transplantation (HSCT) 1980–2010 in denmark or finland. physical fitness was evaluated by the dominant hand grip-strength, timed up-and-go, sit-to-stand, gait speed and two-minute walk tests. Results: Survivors presented significantly lower muscle strength and muscle endurance in the dominant hand-grip strength (median Z-score −0.7, range −4.3–3.9) and sit-to-stand tests (median Z-score −1.5, range −3.5–2.5) compared to age and sex matched normative values of the tests. However, mobility and gait speed were not affected on a group level. The prevalence of frailty (pre-frail 20% or frail 10%) was high among the survivors. In multiple regression analysis, chronic graft-versus-host disease, shorter stature, higher body fat mass and hazardous drinking predicted prefrail/frail status. Common cardiovascular risk factors, such as increased levels of serum triglycerides, higher resting heart rate and diastolic blood pressure, were associated with lower physical fitness. Conclusion: Low muscle strength and a high incidence of frailty were observed in survivors of pediatric HSCT. There is a predominant risk of cardiovascular and metabolic diseases in the long-term.

## 1. Introduction

Allogeneic hematopoietic stem cell transplantation (HSCT) as a therapy for numerous severe hematological disorders is increasing. The burden of treatment-related morbidities in the long term remains considerable [1]. Nearly all children treated with HSCT will experience at least one late effect [2]. Those most frequently observed are endocrine complications such as hypogonadism, impaired fertility, growth delay and metabolic problems, but also other physical and psychosocial problems, such as neurocognitive impairment, cardiotoxicity and second neoplasms have been reported [2,3].

Several treatments affect late outcomes. Anthracyclines, a key component in several childhood cancer treatments, may cause cardiomyopathy [1,2,4]. Total body irradiation (TBI) used in the conditioning regimes prior to HSCT can reduce skeletal muscle mass through endocrine insufficiency [1,2,4]. TBI, busulfan and cyclophosphamide may cause impaired pulmonary function which can limit physical performance [5]. Consequently, many survivors report symptoms of impaired fitness, including exercise-induced shortness of breath, fatigue and reduced participation in physical activity [5,6]. These symptoms may indicate frailty [7].

Frailty is a phenotype generally described in the elderly [8]. The frail phenotype is defined as a clinical syndrome in which three or more of the following criteria are present and pre-frail when two criteria are present: low lean muscle mass, muscle weakness, slow walking, low physical activity or self-reported exhaustion [7,8]. The prevalence of frailty is increased among young adult childhood cancer survivors and is associated with early morbidity and mortality [7]. We have recently reported signs of frailty, premature arterial aging, asymptomatic cardiac dysfunction, increased blood pressure and poor physical fitness among long-term survivors of high-risk neuroblastoma treated with myeloablative regimens [9].

In this study, we investigated physical fitness, physical activity and frailty decades after pediatric allogeneic HSCT in a large population-based cohort of adult male long-term survivors.

## 2. Materials and Methods

### 2.1. Subjects

This study is part of a larger cross-sectional cohort study addressing long-term gonadal function, metabolism, physical fitness, sexual health and quality of life [10,11,12,13]. Male survivors treated with pediatric myeloablative allogeneic HSCT between 1980 and 2010 in Denmark or Finland were eligible to participate if they were ≤16 years of age at HSCT and ≥18 years of age at the time of the study [12]. Patients treated with more than one HSCT were excluded from the study. Of a total of 181 male long-term survivors of pediatric HSCT, 98 (49 Danish and 49 Finnish) men agreed to participate, leading to a participation rate of 54%.

Treatment characteristics were obtained from the medical records. Cyclophosphamide equivalent doses (CED) were calculated as described in Green et al. [14]. Cumulative anthracyclines were calculated as doxorubicin isoequivalents (DIE) using conversion factor 1 for doxorubicin and 0.833 for daunorubicin. A detailed history of present health was assessed with a questionnaire. Smoking and hazardous drinking (weekly intake > 287 g, i.e., ≥24 standard drinks) were reported [15].

At the day of examination, the patients were screened for chronic-graft-versus-host disease (cGvHD) according to the National Institutes of Health criteria as described [12]. Testicular volume was measured using an orchidometer (mL). Mean volume (mL) of the testicles was calculated, when applicable. Body composition was examined by dual-energy X-ray absorptiometry as described [10]. Blood samples were collected between 8:00 a.m. and 10:00 a.m. after overnight fasting and were stored at −80 ºC until analysis. All measurements were performed with standard techniques in the laboratory of the Helsinki University Hospital and Rigshospitalet Copenhagen. All examinations were performed between January and October 2017. The Research Ethics Committee of the Helsinki University Hospital, the Regional Committee of Health Research Ethics, Denmark and the Danish Data Protection Agency, approved the study.

### 2.2. Physical Fitness Tests

#### 2.2.1. Dominant Hand-Grip Muscle Strength by Dynamometer

Sitting hand-grip strength (kg), with the forearm neutral and the elbow flexed 90 degrees, was measured using a Jamar dynamometer (Warrenville, IL, USA) twice with each hand. The highest value attained with the dominant hand was reported. Results were compared to age- and sex-specific normative reference values for grip strength [16].

#### 2.2.2. Functional Mobility by Timed Up-and-Go Test

The patient was seated on a chair with their back against the chair back. A tape mark was placed on the floor three meters from the front edge of the chair. When timing started the patient walked to the tape, turned around, walked back to the chair and sat down with regular pace. Timing (s) ended when the patient was seated again. Results were compared to age- and sex-specific normative reference values [17].

#### 2.2.3. Dynamic Muscular Endurance by Sit-to-Stand Test

The patient was seated on a chair with their feet flat on the floor. The patient was to fully stand up and sit down as many times as possible in one minute in their own pace. Results were compared to age- and sex-specific normative reference values [18].

#### 2.2.4. Gait Speed Test

A 4.6-m (15 feet) walking lane was marked on the floor. The patient walked at his regular pace along the walking lane, and the timing was stopped when the patient crossed the 4.6-m mark with both feet. Gait speed (m/s) was compared to age- and sex-specific normative values of four-meter gait speed in the NIH Toolbox Study [19].

#### 2.2.5. Endurance by Two-Minute Walk Test

A 15.2-m walking lane was marked on the floor. The patient was asked to walk along the lane for two minutes as quickly as possible. The distance walked (m) was compared to age- and sex-specific normative reference values [20].

### 2.3. Physical Activity Assessment

Physical activity was assessed by The Global Physical Activity Questionnaire (GPAQ) developed by WHO [21]. Total metabolic equivalent of task (MET)-minutes per week was calculated using the formula: [cycling/walking MET-minutes/week = 4.0 × cycling/walking minutes × cycling/walking days] + [moderate MET-minutes/week = 4.0 × moderate-intensity activity minutes × moderate days] + [vigorous MET-minutes/week = 8.0 × vigorous-intensity activity minutes × vigorous-intensity days] = total physical activity MET-minutes per week. The WHO recommends physical activity corresponding to a minimum of 600 MET-minutes per week.

### 2.4. Assessment of Frailty

The phenotypes of frail and pre-frail, as originally defined by Fried et al. [8], were modified to this patient cohort. The participants were defined as pre-frail if they fulfilled two and frail if they fulfilled three or more of the following five criteria:

#### 2.4.1. Low Lean Muscle Mass

Body composition was measured by dual-energy X-ray absorptiometry (DXA; Lunar Prodigy Advance fan beam scanner, GE Medical Systems, Madison, WI, USA, Prodigy enCORE software version 16.10.151). Appendicular lean muscle mass (the sum of the lean mass in arms and legs/height, kg/m^2^) of less than 1.5 SD from the mean in the National Health and Nutrition Examination Study (NHANES) was classified as low lean muscle mass [22].

#### 2.4.2. Low Energy Expenditure

The level of physical activity was assessed by the GPAQ questionnaire, as described above. The WHO classification criteria of 600 MET-minutes per week was used as a cutoff level [21].

#### 2.4.3. Slowness

A two-minute walk test result less than 1.5 SD from the age- and sex-specific values was classified as slow [20].

#### 2.4.4. Weakness

Dynamic muscular endurance from the lower extremity was assessed with the sit-to-stand test. A test result less than 1.5 SDs below age- and sex-specific values was classified as weak [18].

#### 2.4.5. Exhaustion

The vitality subscale of the Medical Outcomes Survey Short Form-36 (SF-36) version 2.0 was used to classify exhaustion [23,24]. The cutoff was set at 1.5 SDs below the age- and sex-specific Finnish population mean of 50.

For three patients one of the criteria was missing, and the frailty score was calculated based on four criteria.

### 2.5. Data Analysis

The Mann–Whitney U-test and Fisher’s exact test were used for statistical comparisons between groups. Test scores of physical fitness were transformed into Z-scores based on normative reference values (mean 0, SD 1; higher scores indicate better performance) and Z-scores were used in all data analyses. Physical fitness was compared to expected Z-values with the One-sample Kolmogorov–Smirnov test.

Spearman correlation coefficient was used to study correlations between study outcomes (physical fitness, physical activity and frailty) and transplant-related risk factors, clinical outcomes and laboratory assessments presented in Table 1. All statistically significant results are presented in the text. The parameters that correlated significantly with prefrail to frail health or that were theoretically of interest were further analyzed in a backward multiple linear regression analysis with prefrail to frail health as a dependent variable. Two-sided *p*-values < 0.05 were considered statistically significant. Analyses were performed using the IBM SPSS statistical software (version 27, Chicago, IL, USA).

## 3. Results

### 3.1. Participants

Patient characteristics are presented in Table 1 and disease and treatment characteristics in Table 2.

The survivors had undergone HSCT at a mean age of 9.8 (SD 4.2, range 0.4–16.9) years. Among participants, both hematologic malignancies (75%) and non-malignant diseases (25%) were included. At the time of the study, 28 (29%) patients presented with manifestations of cGvHD; 13 with mild, 12 with moderate and three with severe cGvHD. Three patients were treated with systemic immunosuppressive medication.

### 3.2. Physical Fitness

The measures of the dominant hand-grip muscle strength and sit-to-stand tests were significantly reduced in the HSCT patients compared to the normative reference values of the tests (Table 3). On the other hand, surprisingly HSCT patients walked significantly faster in the timed up-and-go and two-minute walk tests compared to the reference values. Their gait speed corresponded to the reference values.

Of transplant related factors, a higher CNS irradiation dose correlated with lower performance in the dominant hand-grip strength (r_s_ −0.223, *p* = 0.028) and two-minute walk tests (r_s_ −0.303, *p* = 0.003). Longer follow-up time since HSCT was correlated with several domains of poorer physical fitness (timed up-and-go r_s_ −0.422, *p* < 0.001, gait speed r_s_ −0.280, *p* = 0.005 and two-minute walk test r_s_ −0.304, *p* = 0.002). Similarly, older age at examination was correlated with poorer performance (timed up-and-go r_s_ −0.241, *p* = 0.017, two-minute walk test r_s_ −0.215, *p* = 0.035).

Of the clinical outcomes, cGvHD was correlated with poorer performance in dominant hand-grip muscle strength (r_s_ −0.323, *p* = 0.001) and gait speed (r_s_ −0.219, *p* = 0.031). Survivors on testosterone substitution had reduced test results in dominant hand-grip strength (r_s_ −0.250, *p* = 0.013), gait speed (r_s_ −0.265, *p* = 0.009) and two-minute walk tests (r_s_ −0.217, *p* = 0.033). Similarly, survivors on antihypertensive medication had reduced results in the two-minute walk test (r_s_ −0.252, *p* = 0.013). In addition, current smokers had decreased performance in the two-minute walk test (r_s_ −0.242, *p* = 0.017).

Of the clinical assessments, height and testicular volume were positively correlated with dominant hand-grip strength (r_s_ 0.528, *p*< 0.001 and r_s_ 0.511, *p* < 0.001, respectively), gait speed (r_s_ 0.230, *p* = 0.023 and r_s_ 0.365, *p* < 0.001), and the two-minute walk test (r_s_ 0.334, *p* < 0.001 and r_s_ 0.230, *p* = 0.027). Decreased physical fitness in the two-minute walk test was correlated with several cardiovascular risk factors including increased levels of serum triglycerides (r_s_ −0.331, *p*< 0.001), higher resting heart rate (r_s_ −0.251, *p* = 0.013), diastolic blood pressure (r_s_ −0.222, *p* = 0.029) and higher glycosylated hemoglobin A1c (GHbA1c, r_s_ −0.202, *p* = 0.047). Lower performance in the sit-to-stand test was also associated with a higher resting heart rate (r_s_ −0.274, *p* = 0.007). Furthermore, higher fat mass percentage and lower lean mass were correlated with poorer dominant hand-grip muscle strength (r_s_ −0.275, *p* = 0.007 and r_s_ 0.671, *p* < 0.001, respectively), two-minute walk test (r_s_ −0.416, *p* < 0.001 and r_s_ 0.230, *p* = 0.023) and sit-to-stand test (r_s_ −0.197, *p* = 0.053 for fat mass). Survivors with low energy expenditure (MET-minutes <600) had a reduced score in the two-minute walk test (r_s_ −0.216, *p* = 0.034).

### 3.3. Physical Activity

Self-reported physical activity, measured as MET-minutes per week, was not related to previously received treatment regimens. Likewise, follow-up time since HSCT was not associated with physical activity, but younger survivors reported higher activity levels (r_s_ −0.212, *p* = 0.036). None of the clinical outcomes were significantly correlated with self-reported physical activity. Of the laboratory assessments, decreased levels of serum triglycerides (r_s_ −0.249, *p* = 0.013), lower fat mass percentage (r_s_ −0.287, *p* = 0.004) and higher high-density lipoprotein (HDL, r_s_ 0.342, *p* < 0.001) were correlated with higher physical activity.

For the subgroup of survivors who reported the lowest physical activity levels (*n* = 12, 12%), low energy expenditure (<600 MET-minutes per week) was associated with higher levels of serum triglycerides (r_s_ 0.250, *p* = 0.013), GHbA1c (r_s_ 0.200, *p* = 0.048) and fat mass percentage (r_s_ 0.221, *p* = 0.029) as well as lower HDL (r_s_ −0.275, *p* = 0.006). Survivors with low energy expenditure were significantly more likely to need antihypertensive medication (r_s_ 0.240, *p* = 0.017).

### 3.4. Prevalence of Frailty

The prevalence of pre-frailty 20% (20/98) and frailty 10% (10/98) was high among the survivors. Frailty components were reported as low lean muscle mass (36%), self-reported exhaustion (15%), low energy expenditure (11%), slowness (6%) and weakness (45%) among all survivors.

There was only a trend for TBI treated survivors to have more prefrailty or frailty compared to non-TBI treated survivors (Table 1). No correlation was observed between primary hematological disease and the development of frailty. Of the clinical outcomes, cGvHD was significantly more common among survivors with prefrail to frail health. The two survivors who reported hazardous drinking had prefrail status. However, there were similar frequencies of survivors with testosterone substitution, antihypertensive medication and current smokers among the prefrail to frail and non-frail survivors.

Of the laboratory and clinical assessments, frail to prefrail survivors had significantly shorter height, smaller adult testicular volume, higher resting heart rate, lower lean mass and higher fat percentage compared to non-frail survivors. In our cohort, increasing age (r_s_ 0.050, *p* = 0.622) and follow-up time (r_s_ 0.063, *p* = 535) were not associated with frail health among HSCT survivors. Furthermore, total bone mass (kg) was compared between the groups and found to be significantly lower in the prefrail to frail survivors (median 1.1, range 0.8–1.9) compared to the non-frail survivors (median 1.2, range 1.0–1.5, *p* < 0.001).

### 3.5. Risk Factors Associated with Prefrail to Frail Health

A significant regression equation was found, F(4,89) = 8.05, *p* < 0.001, adjusted R^2^ = 0.233. The risk factors included in the backward multiple regression analysis were TBI, cGvHD, current smoker, hazardous drinking, height, adult testicular volume, resting heart rate and fat mass percentage. Significant predictors of prefrail to frail status were higher fat mass percentage (β = 0.260, *p* = 0.008), shorter height (β = −0.240, *p* = 0.016), cGvHD (β = 0.215, *p* = 0.026) and hazardous drinking (β = 0.196, *p* = 0.035).

## 4. Discussion

The present study showed that signs of frail health are prevalent and muscle strength significantly decreased among very long-term survivors of allogeneic HSCT in childhood. Thirty-one percent of the survivors fulfilled two or more components of frail health (low lean mass, low physical activity, weakness, slowness, exhaustion). Chronic GvHD, shorter stature, higher fat percentage and hazardous drinking were shown to be potential factors contributing to frail health after childhood allogeneic HSCT. Poor physical fitness and low physical activity level were associated with older age at examination and cardiovascular risk factors.

The prevalence of the pre-frailty (20%) and frailty (10%) phenotypes in the present cohort were unexpectedly high when considering the young mean age of 28.7 years of the survivors. The prevalence of frailty is known to be 10% among community-dwelling elderly persons above 65 years of age [25]. However, the results are well in line with a previous report of adult allogeneic transplant recipients where the prevalence of frailty was 8% and prefrailty 17% at a mean age of 42 years [26]. The frailty phenotype in pediatric HSCT recipients seems to be somewhat different than described in the aging populations. After childhood allogeneic HSCT, low lean mass was common; muscle weakness and slowness were not. Transplanted children may experience muscle wasting, often compounded by poor nutritional intake, hormonal disruption and decreased physical activity during key periods of development. These factors may contribute to reduced motor competence, physical inactivity, suboptimal lean mass and exhaustion in the decades following HSCT. The present observation suggests a significantly accelerated physical aging especially after childhood allogeneic HSCT.

We have previously reported that high-risk pediatric neuroblastoma survivors treated with TBI and autologous HSCT display frail health and signs of premature arterial aging in early adulthood [9]. In the present study, we were not able to demonstrate a significant association between TBI and frailty or decreased physical fitness. The only treatment related factor that was significantly associated with physical fitness was the total amount of CNS irradiation. However, cardiovascular risk factors were associated with decreased physical fitness, e.g., increased levels of serum lipids, increased resting heart rate and increased diastolic blood pressure. This increases the risk of early onset metabolic and cardiovascular disease warranting continuous cardiometabolic follow-up.

Lower skeletal muscle mass and higher android/gynoid fat ratio has been reported in the present survivor cohort [10]. In the present study, we further showed that sarcopenia associates with decreased physical fitness and is an important criterion for the frail phenotype with 36% of the cohort performing under the clinical cut-off for low lean muscle mass. Furthermore, higher fat body mass was an important indicator of both lower physical fitness and activity. It was also the risk factor most strongly predicting frailty in the regression analysis. Testosterone is closely associated with muscle and fat mass [27]. Consequently, the need for testosterone substitution and decreased adult testicular volume were associated with lower muscle strength, endurance and gait speed among survivors. The endocrine system is a key parameter in poor physical health and frailty because of its many interactions with the whole organism, in particular the brain, the skeletal muscles and the immune system [28]. Testosterone increases the production of muscle proteins by stimulating muscle androgen receptors and by intervening on the Insulin-like growth factor 1 system within the muscle [29]. In a recent meta-analysis, serum free testosterone was suggested as a biomarker for characterizing physical frailty [30]. A parallel decrease of both free and total testosterone is known to occur during physiological aging but is also a hallmark of hypogonadism, which has previously also been reported in the present patient cohort [12]. Several trials have reinforced the beneficial effects of testosterone therapy on muscle strength and symptoms of frailty [31]. After pediatric HSCT, gradual deterioration of Leydig cell function has been reported and a clinically significant reduction may first be detected in early adulthood [12,32]. Therefore, male long-term survivors of pediatric HSCT require special attention regarding symptoms of testosterone deficiency.

Active cGvHD was shown to be a significant contributing factor to frail health and decreased physical fitness after childhood allogeneic HSCT. This observation is in line with a previous report from adult allogeneic HSCT recipients where active cGvHD significantly increased the risk of frailty [26]. The association is expected since cGvHD itself has a significant negative effect on quality of life and symptom burden. In the present cohort, the proportion of current smokers was 20%, and 2% of survivors reported hazardous drinking. These proportions are comparable to the Finnish general population with 16% of 35–44-year-old males being daily smokers and 7% of 17–79-year-old males high-risk alcohol users [33,34]. Despite the potential long-term risks, some survivors continued to engage in these high-risk behaviors that clearly affected their health. Similarly, poor muscle mass and strength as well as obesity were shown to be important risks in this population. The finding that cGvHD, higher body fat mass and hazardous drinking were independently associated with frail health suggests potential modifiable targets for behavioral and clinical interventions to ameliorate frailty. The results overall indicate a need for targeted interventions concerning health information and health promotion for this high-risk population.

A few limitations should be considered when interpreting these results. First, the assessment methods used to define frail health vary substantially between studies affecting the comparability of results. The criteria for frailty can either be defined by different physical tests or by self-reports. In this study, physical tests with age- and sex -specific normative values were used for three of the criteria and standardized self-reports for two (weekly physical activity level and self-reported exhaustion). Our criteria for frailty differed slightly from those used by Fried et al. in an aging population. They used unintentional weight loss to define loss of lean muscle mass, and two questions from the Center for Epidemiologic Studies Depression Questionnaire to characterize exhaustion. It is possible that our choice of different measures than were used in the original study affected results on lean mass and exhaustion. Additionally, the definition for prefrail status varies; some studies use the presence of one or two criteria as the cut-off [8], while others require two criteria [7]. The latter, stricter definition was used in this study. Second, concerning risk factors, more than a fourth of the survivors had testosterone substitution, indicating that testosterone deficiency is a major issue in this patient population. A limitation was that we could not evaluate the treatment effectiveness of the used testosterone substitution since no repeated measurements were undertaken. Third, albeit two national cohorts were collected, the sample size was limited, particularly when the heterogeneity of the sample is considered. However, since 54% of the national cohorts of survivors was enrolled, we believe that this study series represents long-term male HSCT survivors in general. Major strengths of the study include the comprehensive set of physical tests and cardiometabolic health markers.

## 5. Conclusions

Young adult survivors of childhood HSCT have a higher-than-expected prevalence of frailty, suggesting that pediatric HSCT recipients may have accelerated aging. Survivors also presented lower physical fitness and several cardiovascular risk factors, which may increase their risk for cardiovascular and metabolic diseases in the future. Some modifiable predictors for frail health and poor physical fitness were found, namely higher body fat mass, regular smoking and hazardous drinking. These results indicate a need for individual monitoring of motor performance with standardized tests, cardiometabolic follow up as well as active health education in the decades following HSCT. Longitudinal surveillance of survivors is needed to identify those at highest risk and provide targeted interventions like home- or hospital-based exercise programs or active video games to promote physical activity and to prevent or alleviate adverse outcomes associated with frailty in this population.

## Figures and Tables

**Table 1:** Background characteristics of all study participants as well as the pre-frail and frail subgroup compared to non-pre-frail and non-frail participants.

Characteristic	All HSCT	Pre-Frail and Frail Participants	Non-Frail Participants	*p*
	Survivors			
	***n* = 98**	***n* = 30**	***n* = 68**	
Age at examination (years)	28.7 ± 7.3	29.5 ± 8.0	28.4 ± 7.0	0.622
	18.5–47.0	18.7–43.2	18.5–47.0	
Time since HSCT (years)	19.0 ± 7.0	19.8 ± 7.9	18.6 ± 6.5	0.537
	7.7–34.6	8.1–33.4	7.7–34.6	
**Treatment regimens**				
Total body irradiation, *n* (%) ^1^	72 (73%)	26 (87%)	46 (68%)	0.081
Chemotherapy before HSCT,	66 (67%)	20 (67%)	46 (68%)	1
*n* (%)				
Total CNS radiation dose (cGy)	1036 ± 929	1384 ± 1137	842 ± 713	0.181
	0–3600	0–3600	0–3000	
Total gonadal radiation dose (cGy)	987 ± 1009	1208 ± 1067	885 ± 981	0.252
	0–4130	0–4130	0–3600	
Doxorubicin isoequivalent dose (mg/m^2^) ^2^	162 ± 167	150 ± 187	168 ± 158	0.412
	0–750	0–750	0–517	
Cyclophosphamide equivalent dose (mg/m^2^) ^2^	6625 ± 4874	7175 ± 5028	6379 ± 4822	0.247
	0–28,743	2600–28,743	0–23,461	
**Clinical outcomes, *n* (%)**				
Chronic graft-versus-host disease	28 (29%)	14 (47%)	14 (21%)	0.014
Testosterone substitution	25 (26%)	9 (30%)	16 (24%)	0.616
Anti-hypertension medication	12 (12%)	5 (17%)	7 (10%)	0.504
Current smoker	20 (20%)	6 (20%)	14 (21%)	1
Hazardous drinking ^3^	2 (2%)	2 (7%)	0	0.092
**Laboratory assessments**				
Height (cm)	171.6 ± 9.8	166.5 ± 8.6	174.1 ± 9.6	<0.001
	143.2–199.0	143.2–180.2	150.0–199.0	
Testicular volume (ml) ^4^	13.4 ± 7.6	10.6 ± 7.0	14.7 ± 7.6	0.015
	2.0–27.0	2.0–25.0	2.0–27.0	
Triglycerides (mmol/L)	1.9 ± 1.7	2.0 ± 1.2	1.8 ± 1.9	0.07
	0.4–12.4	0.7–5.2	0.4–12.4	
Resting heart rate (beats/min)	73.7 ± 14.3	78.9 ± 13.7	70.3 ± 13.6	0.028
	45.0–122.3	56.0–122.3	45.0–110.0	
Diastolic blood pressure (mmHg)	74.5 ± 11.7	75.5 ± 13.3	74.1 ± 11.5	0.68
	45.3–125.0	52.7–105.0	45.3–125.0	
Systolic blood pressure (mmHg)	127.2 ± 13.3	127.0 ± 14.0	127.9 ± 13.3	0.601
	102.0–174.0	106.0–158.0	102.0–174.0	
High-density lipoprotein (mmol/L)	1.2 ± 0.3	1.1 ± 0.3	1.2 ± 0.3	0.2
	0.5–2.2	0.6–1.9	0.5–2.2	
Glycosylated hemoglobin A1c (%)	6.2 ± 1.4	5.9 ± 0.8	6.3 ± 1.6	0.621
	4.6–13.6	4.6–8.4	4.7–13.6	
Lean mass (kg)	48.1 ± 10.6	40.4 ± 7.8	51.3 ± 10.2	<0.001
	19.7–82.6	20.0–54.9	31.1–82.6	
Fat mass (%)	28.3 ± 7.9	32.2 ± 7.8	26.5 ± 7.4	<0.001
	7.7–45.5	7.7–45.5	10.5–44.6	

Note. Data presented as mean ± SD, range, unless otherwise indicated. Exact *p*-values are derived from the Mann–Whitney U-test or Fisher’s Exact Test. Abbreviations: HSCT, Hematopoietic stem cell transplantation. ^1^ Two patients treated with total body irradiation received a 2 Gy single dose, the rest of the treated patients received 10–12 Gy in 3–6 fractions. ^2^ Data missing for one survivor. ^3^ Weekly intake > 287 g, i.e., ≥24 standard drinks. ^4^ Data missing for four survivors.

**Table 2:** Disease and treatment characteristics of all study participants.

Disease and Treatment Characteristics, *n* (%)	All HSCT
	Survivors, *n* = 98
**Diagnoses**	
Malignancies	74 (75.5)
Acute lymphoblastic leukemia	45 (45.9)
Acute myeloid leukemia	9 (9.2)
Other malignant disease	20 (20.4)
Benign diseases	24 (24.5)
Severe aplastic anemia	13 (13.3)
Immune deficiency syndromes	6 (6.1)
Histiocytic disorders	2 (2.0)
Inherited abnormalities of erythrocyte function	3 (3.1)
**High dose treatment with allogeneic HSCT**	
Donor	
Sibling	47 (48.0)
Related	8 (8.2)
Unrelated donor	43 (43.9)
Stem cell source	
Bone marrow	91 (92.9)
Peripheral blood	4 (4.1)
Umbilical cord blood	3 (3.1)
**Conditioning regimens**	
TBI based conditioning	72 (73)
TBI + Cyclophosphamide	47 (48)
TBI + ARAC: Sytarabine	15 (15)
TBI + Other	10 (10)
Non-TBI based conditioning	26 (27)
Busulfan + Cyclophosphamide	11 (11)
Cyclophosphamide	8 (8)
Other	7 (7)
Acute graft-versus-host disease	69 (70.4)
Grade I	21 (21.4)
Grade II	29 (29.6)
Grade III	16 (16.3)
Grade IV	3 (3.1)
**Late effects**	
Previous growth hormone therapy	26 (26.5)
Therapy for diabetes mellitus	8 (1)

Note. Data presented as *n* (%). Abbreviations: HSCT, Hematopoietic stem cell transplantation; TBI, Total body irradiation.

**Table 3:** Physical fitness and activity of the long-term HSCT survivors (*n* = 97) compared to normative reference values.

	Raw Score		Z-Score ^1^		
Test (Unit)	Median	Range	Median	Range	*p* ^2^
**Physical Fitness**					
Dominant hand grip-strength (kg)	48	20–83	−0.7	−4.3–3.9	<0.001
Timed up-and-go (s)	7.6	4.3–14.2	0.7	−4.5–6.1	<0.001
Sit-to-stand (repetitions)	33	10–78	−1.5	−3.5–2.5	<0.001
Gait Speed (m/s)	0.8	0.4–1.5	0.11	−2.7–5.8	0.549
Two-minute walk test (m)	217.3	98.9–288.8	0.5	−3.4–2.9	<0.001
**Physical activity**					
MET-minutes ^3^	2620.0	0–51,240.0	-	-	-
MET-minutes < 600 min, *n* (%)	12 (12.2%)		-	-	-

^1^ Z-scores were calculated based on normative reference values. For all Z-values, higher scores indicate better performance. ^2^ *p*-values from the One-sample Kolmogorov–Smirnov test. ^3^ Total metabolic equivalent of task-minutes per week.

## Data Availability

The datasets analyzed during the current study are available upon reasonable request.
